# Preclinical Disease or Risk Factor? Alzheimer’s Disease as a Case Study of Changing Conceptualizations of Disease

**DOI:** 10.1093/jmp/jhad009

**Published:** 2023-05-18

**Authors:** Maartje H N Schermer

**Affiliations:** Erasmus MC University Medical Centre, Rotterdam, The Netherlands

**Keywords:** Alzheimer’s, biomarker, disease definition, pragmatic approach, risk factor

## Abstract

Alzheimer’s Disease (AD) provides an excellent case study to investigate emerging conceptions of health, disease, pre-disease, and risk. Two scientific working groups have recently reconceptualized AD and created a new category of asymptomatic biomarker positive persons, who are either said to have preclinical AD, or to be at risk for AD. This article examines how prominent theories of health and disease would classify this condition: healthy or diseased? Next, the notion of being “at risk”—a state somewhere in-between health and disease—is considered from various angles. It is concluded that medical-scientific developments urge us to let go of dichotomous ways of understanding disease, that the notion of “risk,” conceptualized as an increased chance of getting a symptomatic disease, might be a useful addition to our conceptual framework, and that we should pay more attention to the practical usefulness and implications of the ways in which we draw lines and define concepts.

## I. INTRODUCTION

Alzheimer’s Disease (AD) is a slowly progressive neurodegenerative disease, the prevalence of which increases with age. It is considered to be the most common cause of dementia in older age-groups and, because of the decline in mental abilities and personality changes that characterize it, it is much-feared. Over the last decades, scientific understanding of the disease has changed significantly.

In 1984, Alzheimer’s Disease was defined as a dual clinicopathological entity by the National Institute of Neurological and Communicative Disorders and Stroke and the Alzheimer’s Disease and Related Disorders Association workgroup (McKhann *et al.*, 1984). This implied that a diagnosis of AD required both a clinical phenotype—dementia—and presence of specific neuropathological changes: plaques and tangles in the brain tissue. Since these neuropathological changes could only be confirmed postmortem, during life one could only be diagnosed with “*probable* AD.”

Today, the landscape of AD has changed dramatically. AD is no longer seen as a clinical entity, but rather as a prolonged pathophysiological process. At present, one of the leading—although also contested—hypotheses in the AD research community is the amyloid cascade hypothesis. This holds that the process of AD starts with an accumulation of amyloid beta (Aβ) in the brain (plaques), followed by tau-pathology—aggregation of tau-protein in so-called neurofibrillary tangles, leading to neurodegeneration and ultimately cognitive decline (Jack et al., 2010, 2013). The accumulation of Aβ is thought to precede the first clinical symptoms of cognitive decline by about a decade, although it is not necessarily associated with cognitive decline—some post-mortem studies of cognitively healthy people found massive amyloid accumulation (Savva *et al.*, 2009). The first, relatively mild objective cognitive symptoms are labelled as Minimal Cognitive Impairment (MCI); only when cognitive decline becomes so severe as to interfere with daily life does one speak of dementia. Rates of progression from one stage to the next are not fully clear yet, and neither is the predictive value of brain amyloid and tau-pathology (Heister *et al.*, 2011; Wolfsgruber *et al.*, 2017).

Proponents of the Aβ-hypothesis claim that earlier recognition of AD and medical treatment earlier in the process are key to preventing or slowing down cognitive decline. To gather evidence to support this claim, clinical trials need to be performed that focus on early detection and intervention. To align research efforts and to facilitate communication between researchers, new criteria and a new vocabulary for the different stages of AD have been proposed by two different expert groups: the International Working Group for New Research Criteria for the Diagnosis of AD (IWG) and the National Institute on Aging—Alzheimer’s Association (NIA-AA).

In the period 2011–2018 these two groups developed similar but subtly different accounts (see table 1). Both make the shift from understanding AD as a clinical concept toward seeing it as primarily a pathological one. Biomarkers, especially low Aβ and elevated phosphorylated tau in cerebrospinal fluid (CSF), and cortical amyloid and tau on brain PET scans, are used to define the early stages of the disease.^1^ Interestingly, however, the two groups differ in how they categorize abnormal biomarkers in the absence of any clinical symptoms. While the NIA-AA understands this as *preclinical disease*, IWG defines it as *a risk for developing disease*. As Hampel et al. says:

**Table 1.** Simplified representation of the new nomenclature for AD suggested by the working groups.

State	Biomarker presence	NIA-AA disease definition Sperling et al., 2011, 2014; Jack et al., 2012, 2018	IWG disease definition Dubois et al., 2010, 2014	IWG 2016 Dubois *et al.*, 2016
No cognitive impairment	+/- (low level only Aβ abnormal)	Alzheimer’s pathological change (2018)		Asymptomatic at risk for AD
No cognitive impairment	+ (higher levels/ both Aβ and tau abnormal)	Preclinical AD Stage 1-2	Preclinical AD: - Asymptomatic at risk for AD - Pre-symptomatic AD (genetic)	Preclinical AD
Subtle cognitive impairment	+	Preclinical AD stage 3		
MCI	+	MCI due to AD	Prodromal AD	
	-	MCI unlikely due to AD	MCI	
dementia	+	AD dementia	AD dementia	AD dementia

*Note:* MCI, mild cognitive impairment; AD, Alzheimer’s Disease (adapted from Schermer and Richard, 2019).

The IWG group considers the presence of brain Aβ accumulation in the absence of clinical features in the sporadic population to be indicative of an “at risk” group. In contrast, the NIA-AA group considers such individuals to indeed already have preclinical AD, suggesting that in time they would develop cognitive decline and the clinical dementia syndrome. This presents a fundamental hypothetical and conceptual difference of the two approaches. (2014, 431).

This situation raises the question: which of these two conceptualizations is the most defensible, given our concepts of health, disease, and risk? Apart from being interesting in itself, the reconceptualization of AD thus offers a great case-study to “test” our current medical-philosophical theories and conceptual frameworks—can they elucidate practical scientific and clinical questions with regard to definition and classification of pre-disease and risk? Making such an analysis contributes to the growing body of work investigating how concepts of disease “fit” with clinical and research practices (e.g., De Vreese, 2017; Reid, 2017).

The goal of this article is, first, to gain a better understanding of the categories of “preclinical AD” and “being at risk for AD.” Should these asymptomatic states, in which evidence for Aβ accumulation and tau-pathology is present, be regarded as disease, or as a risk factor, or something else? The second goal is to diagnose some of the problems that current medical-philosophical theories run into when trying to conceptualize health, disease, and risk in light medicine’s move toward ever earlier detection of pre-disease states, of which AD is just one example (Arias *et al.*, 2017).

I will start by showing the confusion among AD researchers themselves and their attempts to build a coherent framework to conceptualize AD and various “pre-” states. Next, I will ask how two prominent theories of health and disease would classify the condition of someone who is asymptomatic but has abnormal AD-biomarkers: healthy or diseased? Since it turns out these theories have little to say about the condition of being at-risk—a state that might be seen as something in-between health and disease—section IV will further examine the notion of being at-risk for AD and make a plea for a more pragmatic approach. The last section will draw some conclusions with regard to the shortcomings of current theories as illustrated by the developments in the AD research field, suggest a more pragmatic direction with regard to the philosophical work that needs to be done to get a better grip on notions of risk and pre-disease, and finally propose to use “at risk of symptomatic disease” as a new category in this field.

## II. NEW CONCEPTS AND VOCABULARY FOR AD

The two groups that worked on the reconceptualization of Alzheimer’s disease, the IWG and the NIA-AA, although partly comprised of the same people, worked separately and implicitly seem to hold slightly different concepts of disease. They sometimes use the same terms to describe different phenomena or, vice versa, use different terms to describe the same thing. Due to these complexities, the new lexicons have rightly been referred to as a “tower of Babel” (Giaccone *et al.*, 2011).

In a series of articles NIA-AA define AD as “encompassing the underlying pathophysiological disease process, as opposed to having ‘AD’ connote only clinical stages of the disease” (Sperling *et al.*, 2011, 4). This is an important deviation from previous use of the term, in which “AD” was only used to refer to dementia. According to these new guidelines, however, AD should be understood as a disease-continuum (Jack, 2011; Sperling *et al.*, 2014). This implies that according to NIA-AA, AD can be present without any symptoms—this is called the preclinical stage of AD, or in short “preclinical AD.” The next stages are the “symptomatic, pre-dementia phase” (also: MCI due to AD) and the “dementia phase of AD” (also: AD dementia). “Preclinical AD” can be subdivided in three sub-stages, the first two of which includes persons without cognitive symptoms but with biomarker evidence of “AD pathology,” that is Aβ accumulation, tauopathy, and neurodegeneration. The third substage involves “subtle cognitive decline.”^2^

The IWG, in their articles from 2010 and 2014, state that AD is a clinicopathological entity and that symptoms are crucial for making a diagnosis (Dubois et al., 2010, 2014). They do distinguish a related asymptomatic state; however: preclinical AD. According to IWG, this category should not be called a disease because of the absence of clinical symptoms. It includes “pre-symptomatic AD,” which applies to individuals with a rare autosomal dominant genetic mutation that will definitely lead to cognitive decline in the future, and “asymptomatic at risk for AD.” I will focus here on the latter category, which (like the NIA-AA category of “preclinical AD”) includes people with abnormal biomarkers. For persons who fall in this category, it is uncertain whether they will develop cognitive decline in the future, although they are believed to be at a higher risk of doing so, and therefore they are called “at risk for AD.” This classification-system gives rise to the somewhat confusing situation that a person can be labelled as having “preclinical AD-at risk of AD” and thus apparently as both *having* a preclinical disease and *being at risk for* that disease at the same time (cf. Sperling *et al.*, 2014). What is meant here—but not spelled out—is that the first “AD” refers to the presence of biomarkers indicating AD *pathology*, while the second refers to *clinical*, symptomatic, AD. This nicely illustrates the confusion in the field about whether “preclinical AD” should be considered a disease or a risk factor for disease.

In a later paper, from 2016, leading proponents of the IWG-criteria shift their position by making a slightly different distinction than before between “preclinical AD”—an asymptomatic phase of Alzheimer’s disease in which biomarker evidence for AD pathology is present—and “asymptomatic at risk for AD,” where there is some biomarker evidence but insufficient to diagnose preclinical AD (Dubois *et al.*, 2016). This shift implies that according to IWG, AD can now be diagnosed without the presence of symptoms, just as the NIA-AA already stated earlier. “Preclinical AD” is now explicitly called a disease, and a new category of “asymptomatic at risk for AD”—an at-risk state, not a disease—is created (Dubois *et al.*, 2016).

In this later work, the IWG are also clearer about the distinction they make between asymptomatic and symptomatic disease as two sub-categories of “disease.” They now consistently speak of clinical disease versus preclinical, asymptomatic disease. The NIA-AA proposals of 2011 and 2014 also tried to be clear in this respect by distinguishing between AD-P (i.e., pathophysiological process) and AD-C (i.e., clinical). In their latest 2018 paper, however, they again complicate things by proposing a distinction between “Alzheimer’s pathological change” (if only evidence for Aβ accumulation is present) and “Alzheimer’s disease” (when there is also evidence for tau-pathology). Both fall under the umbrella notion of “Alzheimer’s continuum”—a term that confusingly lacks any reference to disease or pathology (Jack *et al.*, 2018).

As this brief exposition illustrates, the scientific discussion about the reconceptualization of AD is marked by significant conceptual and terminological confusion.

At the same time, the whole project raises important and interesting theoretical questions about the conceptual relationship between clinical symptoms, pathological processes and the notion of “disease.” Moreover, it raises questions about the relationship between asymptomatic or preclinical disease and risk factors or at-risk states for disease. Different theoretical outlooks on health and disease appear to underly the work of the two groups, although this remains mostly unarticulated.

In the following section, I will explore how philosophical theories of health and disease would look upon the presence of abnormal biomarkers in the absence of any symptoms. Should this indeed be understood as disease, as the notion “pre-clinical AD” suggests?

## III. IS “PRECLINICAL AD” A DISEASE?

To answer the question whether “preclinical AD”—abnormal biomarkers in the absence of symptoms—should be understood as a disease, I will analyze this question from the point of view of two well-known and influential theories: Boorse’s Bio Statistical Theory of health (BST) and Nordenfelt’s Holistic Theory of Health (HTH) (Boorse, 1977, 1997, 2014; Nordenfelt, 1987, 2007, 2013).

### The perspective of Boorse’s BST

According to the BST, “health” is the statistically normal biological functional ability of all parts of the organism, contributing to its survival and procreation. Since health and disease are seen as mutually exclusive, disease is defined as any disturbance of health, that is, any statistically subnormal functioning of one or more parts of the organism. In Boorse’s own words: “A disease [later, pathological condition] is a type of internal state which reduces health, that is, reduces one or more functional abilities below typical efficiency” (2014, 684). In the longstanding debate on the question of whether or not health and disease are value-free concepts, Boorse’s position is paradigmatic for the naturalist view that they are. His BST considers pathophysiological processes to be the objective, natural essence of disease.

So, what would the BST say about “preclinical AD?” If we agree that diseases are characterized by dysfunction—that is, by physiological processes that work with statistically subnormal efficiency in a way that ultimately threatens survival or reproduction—then whether preclinical AD should be called a disease depends on the answer to the question of whether there is dysfunction. Do the first signs of Aβ accumulation in the brain already count as species-subnormal part-functioning? This, in turn, depends on which function or functions we should take into account here. If one considers the “species typical function” of the affected brain areas to be cognitive functions—memory, attention, language, executive functions—then it might seem that there is no dysfunction in the preclinical stage, since this stage is defined as the presence of biomarkers in the *absence* of any cognitive symptoms. Boorse himself, however, has emphasized time and again that his theory represents a pathologist’s view, not a clinician’s, and he says: “to the pathologist, any process causing cellular dysfunction, no matter how local, is pathological” (1997, 46) Moreover, “pathologists also recognize micro dysfunctions as pathological” (Boorse, 1997, 48). Therefore, even dysfunctions at a cellular or sub-cellular level, such as abnormal clustering of Aβ protein, should be considered pathological conditions. Boorse adds that “since cellular pathology can be very local -…- many types of pathology will never cause illness unless aggregated” (1997, 47), hereby stressing the distinction he makes between disease and illness.^3^ According to the BST, it is not necessary for a condition to unavoidably or even likely lead to illness, in order for it to be called pathological—some conditions are pathological (i.e., diseases) without ever becoming symptomatic. As Boorse writes: “It is not true that all disease or pathology is of a type ‘likely’ to cause clinical illness” (1997, 47). This also implies that even if Aβ depositions—or tauopathy or neurofibrillary tangles—do not always or not necessarily lead to symptomatic disease and dementia, they would still be considered as pathological by the BST.

One might wonder how such a small and local pathological condition would threaten survival or reproduction—one of Boorse’s other criteria for disease. However, this criterion should not be taken to mean that individual survival or procreation is threatened directly, but rather that the function of the disturbed physiological processes consists in its ultimate contribution to the functioning of the entire organism. In Boorse’s view the structure of organisms shows a means-end hierarchy with goal-directedness at every level.^4^ Small and local incidences of subnormal functioning, such as Aβ accumulation, may therefore be considered pathologic without them ever becoming symptomatic for the individual, or threatening her well-being or survival: “At bottom, disease is a pathological, not a clinical, concept, in that all sorts of subclinical pathology can exist without, or before, clinical manifestations” (Boorse, 1997, 48).

In summary, according to the BST, it seems that preclinical AD must indeed be called a disease or a pathological condition. The only caveat here is that it is unclear whether AD pathology is actually “statistically subnormal.” If we stick to Boorse’s own idea of the reference class, it turns out that AD pathology is not abnormal at all in certain age-groups. Above 80 yrs., >60% of the population has AD pathology (Jack *et al.*, 2018) and hence this should perhaps not count as a pathological condition after all for this age group. However, if we abandon the idea of age-related references classes and adopt “adults” as one single reference class—as Boorse (2014) has suggested we might—AD pathology must count as disease. This latter view is further strengthened by the recognition of Aβ plaques and neurofibrillary tangles as pathological by the professional group of pathologists (e.g., Braak and Braak, 1991; Mirra *et al.*, 1991), since according to Boorse “the considered usage of pathologist” that is, “their considered judgments of individual conditions as normal or pathological” can count as a benchmark for the BST (2014, 712). The only problem with this approach is that, as the IWG has remarked, if we would include all pathological lesions as an instance of AD pathology “the prevalence of the disease, based on neuropathologic evidence, would excessively increase as almost all post-mortem assessment >70 years shows both types of AD brain lesions” (Dubois *et al.*, 2016, 298). This consequence is something that pathologists themselves—Boorse’s own “golden standard”—found undesirable. Therefore, they have set a threshold for the number of lesions needed to establish the diagnosis of AD (Dubois *et al.*, 2016).

### The perspective of Nordenfelt’s HTH

Nordenfelt’s holistic theory of health (HTH) defines health as “being in a bodily and mental state that is such that one has the ability to realize all their vital goals” (2013, 24). Disease is understood as any bodily state or process that tends to reduce health, that is, a state that tends to prevent people from realizing their vital goals. Nordenfelt considers the ability to attain vital goals, and hence the impact of clinical symptoms on the lives of people, to be the fundamental aspect of disease. This implies that for Nordenfelt (2007), “illness,” and not “disease” is the primary notion. Illness here refers to a state in which the person is unable to realize their vital goals due to internal bodily processes or injuries. Illness is the effect of disease, whereas disease refers to the physiological basis of the inability to attain goals, that is, to the causal physical processes that are central to Boorse’s account.

What does this mean for the status of preclinical AD? Clearly in the preclinical stage, when there are, by definition, no symptoms, there is no impact on the ability to attain vital goals, and hence there is no illness. But does this mean there is no disease, according to Nordenfelt? This appears an overly simplistic conclusion, since it would rule out the possibility of recognizing asymptomatic diseases *tout court*. This is not what Nordenfelt intends. He says that not all instances of disease need to lead to illness, but that something is a disease only when it “tends to cause illness in most cases when it is instanced” (Nordenfelt, 1987, 106). In the case of preclinical AD, one might therefore argue that there is disease in an early stage, if (and only if) the AD-pathology—as represented by Aβ and tau biomarkers—will eventually lead to illness in most cases. This would imply that to justifiably call preclinical AD a disease, persons in whom this condition is present would eventually have to develop cognitive impairments to such an extent that they will interfere with the ability to attain vial goals in a majority of cases.^5^

Nordenfelt explicitly recognizes that disease processes can progress over time, and may only lead to symptoms—and hence illness—in later stages. It is not clear, however, how Nordenfelt looks upon the time lag between the onset of disease and the onset of illness, and whether he would recognize conditions as disease if they tend to cause illness only after several decades. Moreover, it is not fully clear whether “tends to cause illness” should be understood as referring to the likelihood that the individual patient will actually come to suffer symptoms, or to the likelihood that illness would eventually occur if only the patient lived long enough. Especially with AD this is relevant, since many people die with AD-related pathological changes in their brain but without having any serious cognitive symptoms (Savva *et al.*, 2009). If they would not have died from other causes, however, they would most likely have developed cognitive decline—or so the amyloid hypothesis claims.

Interestingly, processes that only cause ill health in some instances but not in most cases are also accounted for in Nordenfelt’s theory—he calls them “risk factors” (1987, 106). So, according to the HTH, depending on how frequently the condition of “preclinical AD” actually leads to cognitive impairments that compromise the ability to attain one’s vital goals, it should be called either a disease or a risk factor.

### NIA-AA and IWG proposals in light of the BST and HTH

If we take another look at the NIA-AA and IWG proposals regarding to the concept of preclinical AD, it is apparent that the NIA-AA proposal is mostly in line with a Boorsian account of disease, whereas the IWG seems to lean towards a Nordenfeltian view. According to NIA-AA, asymptomatic presence of biomarkers should already be considered a disease-state because pathological changes are seen as the core of disease. Contrary to this view, the early IWG papers (Dubois et al., 2010, 2014) state we should speak of disease only when symptoms are present, that is in what they call the prodromal stage. In line with the basic tenet of the HTH, they consider the clinical symptoms and their impact on patient’s lives as the essence of disease. The way in which they distinguish, especially in their later 2016 article, between asymptomatic disease and an at-risk state, is also in line with Nordenfelt’s reasoning, as will be discussed further on.

## IV. PRECLINICAL DISEASE, OR RISK FACTOR?

In the previous section, I discussed the appropriate classification of a state in which there are no cognitive or other symptoms, but Aβ accumulation and/or tauopathy are present in the brain, as evidenced by CSF or PET biomarkers. According to NIA-AA and the BST this should be understood as an early, asymptomatic, preclinical phase of Alzheimer’s disease; according to IWG and the HTH it should be understood as an at-risk condition, at least if the likelihood of developing into clinical symptoms is relatively low. While neither the BST nor the HTH have elaborated much on the notion of risk in relation to health and disease, this appears to be an increasingly important category in clinical and preventive medicine. In this section, I will therefore focus specifically on the notion of being at-risk for AD. What does it mean to “be at risk,” or to “have a risk factor,” as opposed to “having a disease?”

As a preliminary, it must be noted that in the clinical and research literature about AD it often remains unclear what exactly “at risk for AD” means because when the notion “at risk for AD” is used in the NIA-AA and IWG articles it is not always spelled out what “*AD*” refers to. Sometimes, it refers to developing Alzheimer’s *Disease*, defined as pathophysiological change. Mostly, however, it refers to Alzheimer’s *Dementia*, so, to clinical, symptomatic disease. In that case, “being at risk for AD” means having a higher-than-average chance (compared to either the general population, or people from one’s age group) to become clinically demented. As explained earlier, this is how the IWG 2014 category of “preclinical AD-at risk of AD” should be understood: as having abnormal biomarkers indicating AD pathology, putting one at-risk for developing AD *dementia* or *clinical* AD. The latest NIA-AA framework (Jack *et al.*, 2018) also uses the term risk as indicating the risk of subsequent cognitive decline and development of clinical symptoms; they actually agree with IWG that having preclinical AD means being at risk for cognitive decline and the development of Alzheimer’s dementia.^6^ The real disagreement thus does not lie in the question of whether these specific pathophysiological changes in the brain are a risk factor for developing clinical dementia—but in the question whether they should be labeled as asymptomatic *disease*, or as a *risk factor* for developing symptomatic disease. Actually, there would be no contradiction in doing both at the same time. Understanding asymptomatic pathology as a risk factor—in the sense of increasing the likelihood—for developing symptomatic disease makes perfect sense in many cases. It does, however, constitute a somewhat different use of the notion “risk factor” than, for example, in epidemiology, where risk factors are often determined based on mere correlation or on more distant causal factors.

### Asymptomatic presence of biomarkers as risk factor - IWG

The distinction that Dubois et al. make in their 2016 article is more precise in spelling out their view on the difference between asymptomatic disease and an at-risk state. They define “risk” explicitly as the probability for a patient to develop the clinical symptoms in the rest of his or her lifetime. When this risk is high, they speak of “disease” (preclinical AD); when this risk is low, they speak of “at-risk” for AD:

Based on the high-risk or low-risk dichotomy for a further progression to clinical AD, we propose to consider the terms of “preclinical AD” when the risk is particularly high (e.g., both Aß and Tau markers beyond pathologic thresholds) and that of AR-AD when the evolution to a clinical AD is less likely or still needs to be determined (only one pathophysiological marker considered abnormal). (Dubois *et al.*, 2016, 296)

Furthermore, the authors state that this distinction is in a sense arbitrary, as the distinction between high and low risk is not an objective fact but a matter of decision. They even suggest the threshold could be tailored to the study in which it is used.

So, the IWG consider that “AD can exist and can be recognized before the onset of cognitive symptoms when there is little doubt about progression to clinical disease over a short period” (Dubois *et al.*, 2016, 297) If this likelihood of progression to clinical disease is deemed lower, they speak of a risk factor instead of a disease. As mentioned above, this is in line with Nordenfelt’s approach to disease and risk. For Nordenfelt, the likelihood that a pathological state will lead to symptomatic disease (i.e., illness), in a way that will interfere with the person attaining their vital goals, determines whether we should speak of disease or risk factor. Nordenfelt speaks of “in most cases,” while Dubois *et al.* (2016) speak of “high” or “low” risk, but neither gives a more precise indication of what this means. Wherever the line is drawn exactly, however, we can conclude that from this perspective the asymptomatic presence of—some level of—AD biomarkers, if it does not convey a high risk of progressing to cognitive decline and dementia, does not constitute a preclinical disease state but rather an at-risk condition. These—lower levels of—biomarkers should be considered as risk factors in this view, not as indicative of the presence of a disease.

### Asymptomatic presence of biomarkers as “risk-based disease?”

Interestingly, although the NIA-AA workgroup generally follows a Boorsian view on disease as essentially a pathological state or pathophysiological process, in their latest framework they raise confusion by comparing AD biomarkers with conditions such as osteoporosis, hypertension and hypercholesterolemia (Jack *et al.*, 2018). They thus compare AD with conditions that are often seen as risk factors, rather than as diseases, and suggest that AD is similar to these conditions with respect to the relation between biomarkers and the concepts of risk factor and disease—which is not in line with the rest of their work in which they define biomarkers as being the disease itself.

In making this comparison, the workgroup refers to what Karlawish (one of the members of the NIA-AA) has called “desktop diseases.”

Desktop diseases are discovered when studies show a factor (e.g., blood pressure) is associated with a negative health outcome (e.g., stroke), and then a clinical trial shows that an intervention affecting that risk factor reduces the risk of that outcome event associated with negative health outcomes that can be positively influenced by intervention in the factor. (Karlawish, 2010, 2061)

This notion is identical to the notion of “risk-based disease” as defined by Schwarz (2008).

As Schwarz (2008) points out, next to notions of absolute and relative risk for contracting or developing disease, there is a third way of understanding the notion of disease-risk, namely as modifiable risk. In risk-based diseases such as hypertension, or hypercholesterolaemia, the notion of elevated risk “refers to the existence of risk that can be lowered, rather than any comparison with the mean for the population” (Schwarz, 2008, 323). He calls such conditions, based not on statistical deviance or pathological dysfunction, but merely on the modifiability of the risk they convey “risk-based diseases.” The latest version of the NIA-AA framework suggests—although just in one single section—that in their view, AD is such a risk-based disease in the exact sense that Schwartz has defined it: “Other areas of medicine have used this approach to define pathologic processes using biomarkers, for example, bone mineral density, hypertension, hyperlipidaemia and diabetes are defined by biomarkers. *Interventions modulating these biomarkers have been shown to reduce the likelihood* of developing fractures, myocardial and cerebral infarctions” (Jack *et al.*, 2018, 538, emphasis mine).

According to Schwarz (2008), however, conditions like hypercholesterolemia and hypertension are not real diseases in a Boorsian sense, because there is no “dysfunction” in the cholesterol or blood-pressure regulating systems.^7^ They are both, therefore, best understood as non-pathological conditions that are risk factors for true diseases such as heart attacks or cerebrovascular incidents; they are not diseases in themselves. Boorse agrees on this point with Schwarz that “risk factor and disease are two separate categories badly confused in contemporary risk-based medicine” (Boorse, 2014, 703).

Although in most of their work NIA-AA state that Aβ and tau biomarkers are indicative of pathological processes that are going on in the brain, in the above statement they seem to imply that these biomarkers are in fact risk factors (for developing cognitive impairments) and that the risk can be mitigated by intervening on these biomarkers. This raises the question whether Aβ and tau biomarkers in CSF or PET are just like low bone mineral density, hypertension or hyperlipidaemia: not pathological in themselves, but risk factors for pathology. Above, I have argued that on my interpretation of Boorse’s theory, Aβ deposits and tauopathy *do* count as pathological. But since the concept of dysfunction is complex and contested, and the actual (patho-)physiological processes in AD are not fully known, other interpretations may well be possible. It might be argued that what matters is cognitive dysfunction caused by neurodegeneration, and that the presence of Aβ and tau biomarkers constitutes a non-pathological modifiable risk factor for this. However, contrary to the risk-based diseases mentioned, AD biomarkers are not successfully modifiable.^8^ Until there is sufficient evidence that some treatment or medication will lower both the biomarkers as well as the incidence of cognitive decline and Alzheimer’s dementia, AD biomarkers are not risk factors in the sense in which this term is used in the context of risk-based disease. Moreover, since this statement of the NIA-AA is not in line with the rest of their work that emphasizes, time and again, the underlying pathophysiological processes to be the essence of AD, I believe it can be considered a slip of the pen.

### Asymptomatic presence of biomarkers and the “line drawing problem”

Yet another way to approach the distinction between (preclinical) disease and risk factor is by understanding it as an instance of the line drawing problem (Schwarz, 2007, 2008, 2017; Rogers and Walker, 2017). As discussed previously, from a Boorsian perspective the exact line between diminished functioning and real dysfunction or between pathology and normal variations is hard to draw. According to Boorse, this distinction merely depends on statistical normality and hence on the prevalence of a certain trait or level of function. As noted, this approach leads to problems in cases where pathological changes are ubiquitous, as is the case with AD pathological lesions in older age-groups.

Although Schwarz holds a mainly Boorsian disease concept, he departs from Boorse in the way he argues the line between low-normal function and dysfunction should be drawn. This should not be done based on mere “statistical normality” but on a combination of prevalence of a specific trait or level of functioning in combination with its negative consequences (Schwarz, 2007, 2017). Hence, a trait or a specific level of functioning may not be statistically very abnormal (e.g., may be present in 15% or 20% of the population) but if it causes important negative consequences, it should be considered disease. Likewise, a trait or function that is not uncommon but rarely leads to negative consequences, should not be considered as such.

Schwarz agrees that there are no exact guidelines for settling the line-drawing issue in borderline cases, and that judgments in some cases may go either way. In the case of ductal carcinoma in situ (DCIS)—the most commonly diagnosed form of breast cancer—he concludes that according to his approach this is not a disease (Schwarz, 2014). Rather, in his view, it is a normal variation that is a risk factor for breast cancer. Along those lines of argument, at least some asymptomatic biomarkers for AD should probably not be called disease either, but risk factors. The proposal of Dubois *et al.* (2016) to distinguishing low-risk and high-risk asymptomatic biomarker conditions, is fully in line with this theoretical approach, since it proposes drawing the line between risk and disease by looking at both prevalence and chance of negative consequences. Interestingly, this indicates that a mainly Boorsian approach—like Schwarz’—and a Nordenfeltian approach may not be that different in their practical consequences after all. Both may end up drawing a line between risk factor and asymptomatic disease based on the magnitude of the probability of developing symptomatic disease.

### Complex disease as a matter of degrees

A challenging question that the previous reflections confront us with, is whether the binary distinction between health or normality on the one hand, and disease or pathology on the other, is still viable. Giroux claims that: “Boorse relies heavily on the possibility of making a clear distinction between a disease and its causation. Yet, it could be asked whether such a distinction can be drawn so easily” (2015, 187). This is especially true for complex and progressive pathological processes like Alzheimer’s disease, as we have seen. According to Giroux, no strict distinction should be made between the normal and the pathological, or between internal risk factors and actual disease. Rather, we should adopt a comparative notion of health: individuals can be more or less healthy to the degree that their physiological systems function more or less efficiently. This appears in line with the idea of an Alzheimer’s continuum (Jack *et al.*, 2018).

However, to problematize the idea of a simple dichotomy between health and disease even further, it should be noted that the idea of one single Alzheimer continuum, solely dependent on amyloid and tau biomarkers, seems overly simplistic. According to many researchers, what is commonly called Alzheimer’s dementia is best understood as a multifactorial syndrome, in which numerous distal and proximal causal factors interplay, alongside “normal” physiological changes related to ageing. As Richards and Brayne state: “In older age groups Alzheimer’s disease seems to be a diffuse clinical syndrome representing the gradual accumulation of multiple pathologies, arising from multiple interlocking risk factors over the life course” (2010, 865). Reid (2017) has rightly warned against singling out one pathogenic factor or pathway for disease at the cost of ignoring other relevant factors such as variations in the natural course of the disease, resistance and compensation on the part of the organism and the probability that there are multiple causal pathways to the same (biological) functions. Singling out an amyloid and tau based one-directional continuum as *the* model for understanding Alzheimer’s dementia fails to take other causal - and mitigating - factors into account and thus gives a wrong picture with regard to dementia risk (cf. Sweeney *et al.*, 2019). Complex multicausal models will likely be better able to predict risk of symptomatic disease—that is, dementia—than does a single pathway model. Alzheimer’s disease is therefore a good example to show that the distinction between health and disease is not dichotomous, but neither can it just be replaced by a simple continuum along one single pathophysiological dimension.^9^

### Pragmatic line drawing

Given this complex picture of health and disease, where health may “grade into disease, without there being an obvious point of transition” (Schwartz, 2017, 494), one might ask whether and why we should aim to draw lines at all. Why do we need to make distinctions between health, disease, and risk?

I believe this is a very important question that may receive a different answer depending on the particular context in which it is asked. We should ask for the purpose of drawing lines in any particular instance, since *how* to draw a line is intimately connected with *why* we want to draw a line in the first place. Line-drawing is useful when it helps to solve some kind of theoretical or practical problem, for example for purposes of research, in order to be able to study disease progression or to determine who should be included in a clinical trial. It may also be necessary to draw lines in order to determine whom to diagnose, treat or follow-up in a clinical setting. Rogers and Walker (2017) and Schwarz (2017) have also recently argued in favor of drawing stipulative lines for practical purposes, depending on the sort of problem that a clear demarcation between disease and non-disease is supposed to solve in any particular case. Others have also argued for more practice-related definitions of disease, that are relative to particular purposes or specific contexts (e.g., Nordby, 2006; De Vreese, 2017; Walker and Rogers, 2018). I am sympathetic to their proposals and have argued elsewhere for a somewhat similar pragmatic approach to conceptualizing health and disease (Schermer and Richard, 2019).

If we accept that health and disease are not two neatly separated states, but that there are various continuities, then classifying certain (temporary) states as “at risk,” or “disease,” is a matter of decision and of drawing the boundaries “in a reasonable place” (Schwarz, 2017). What is “reasonable” should, in my view, be guided by practical considerations, for example by considering how we can draw lines in such a way that it provides meaningful preventative options, while minimizing the harms of overdiagnosis and overtreatment (cf. Doust *et al.*, 2020). Another consideration to take into account would be the unintended and undesirable psychological and societal effects of certain classifications. For example, diagnosing people with “preclinical Alzheimer’s disease” may have more negative psychological effects on people than explaining they are at increased risk of developing Alzheimer’s dementia in the future. It may also lead to stigmatization and social exclusion or even have a nocebo-effect (Schermer and Richard, 2019). Therefore, the notion of “risk factor,” when explicitly conceptualised as an increased chance of getting a *symptomatic* disease, may be a useful addition to our conceptual framework that will help potential patients to better understand their condition, without inducing undue fear.

## V. CONCLUSIONS

Alzheimer’s Disease provides an excellent case study to investigate new and emerging conceptions of health, disease, pre-disease, and risk. A first conclusion that can be drawn is that the notion of “risk” is underdeveloped in two classic medical-philosophical theories of health and disease, the BST and the HTH, while this notion is becoming ever more important in contemporary medicine. Both theories have insufficiently incorporated new scientific insights showing the complexities of pathophysiological processes. Both fail to take the temporal dimensions of pathophysiology into account and appear to see “disease” primarily as a state, rather than as a process that develops over time. Moreover, both implicitly appear to assume that diseases are mono-causal rather than multifactorial complex pathological networks. Hence, they tend to see health and disease as two clearly demarcated, mutually exclusive states. This does not seem in line with current scientific understanding of disease processes.

As the Alzheimer case illustrates, progress in medical science enables us to make visible ever smaller lesions and to find disruptions in functioning at the molecular and sub-molecular level. The chances for people with those micro-pathologies of ever developing clinical symptoms is less than 100%, but higher than the chances of people without this specific pathology. This implies that in many—perhaps in most—of these cases disruptions will never lead to overt illness, raising the question whether it is useful to put all these micro-pathologies in one basket with serious and even life-threatening diseases. It appears more apt to challenge binary ways of thinking in which health and disease are mutually exclusive categories and instead to understand them as complex, multi-dimensional continua between complete health and symptomatic disease. To demarcate specific states within such a field, a criterion of practical usefulness should be used, in the awareness that classifying such (temporary) states is a matter of drawing boundaries in “reasonable places,” and “reasonableness” is related to the practical purpose of line-drawing in specific circumstances. This pragmatic approach will help us to make sense of the blurring boundaries between health, disease and risk, created by the multi-causal process-view of disease.

Finally, from the point of view of potential patients, the magnitude of the probability of getting symptomatic AD, especially dementia, is what matters most. For purposes of communicating with potential patients or research-subjects, therefore, the notion of risk factor should be specified as risk of *symptomatic* disease, that is, a risk of getting Alzheimer’s dementia, and the notion of preclinical disease (suggesting a binary distinction between health and disease) should best be avoided. The concept of “risk of symptomatic disease”—as distinct from a risk of getting some asymptomatic pathology—is a useful addition to our conceptual framework and better captures what is at stake for potential patients than the notion of preclinical disease. This does not preclude that for specific research-purposes other concepts and different lines may be more useful.

## Footnotes

1 Levels of Aβ and P-tau in CSF are on a continuum and cut off points have been defined to mark them as normal or abnormal. Amyloid plaques and tau tangles on PET scans are considered abnormal (although they can exist in cognitively healthy people) and can be graded to level of severity. Whether there is a direct causal link or only a correlation between these various biomarkers and clinical symptoms is not clear, and neither is it fully clear what the predictive value of these biomarkers is for progression of the disease (Scheltens *et al.*, 2016).

2 “Subtle cognitive decline” here presumably refers to subjective decline. Interestingly, they also discern a “substage 0: Older individuals with no biomarker evidence of AD pathology” (Sperling, Karlawish, and Johnson, 2013, 2). It is unclear why persons with no indication of disease whatsoever, should be categorized as having “preclinical AD.” This is probably the reason that in later papers, this category does not re-appear.

3 According to Boorse, illness is a sub-category of disease: “A disease is an illness only if it is serious enough to be incapacitating, and therefore is: 1. Undesirable for its bearer; 2. A title to special treatment; and 3. A valid excuse for normally criticizable behavior” (1975, 61). In later work, Boorse abandoned this usage, and uses different terms, such as “clinical disease” (1997, 48).

4 “Individual cells are goal-directed to manufacturing certain compounds; by doing so they contribute to higher-level goals like muscle contraction; these goals contribute to overt behavior like web-spinning, nest-building, or prey-catching; overt behavior contributes to such goals as individual and species survival and reproduction. What I suggest is that the function of any part or process, for the biologist, is its ultimate contribution to certain goals at the apex of the hierarchy” (Boorse, 1977, 556).

5 What “most cases” means, exactly, is not clear from Nordenfelt’s writings.

6 NIA-AA mistakenly claims that their use of the term risk differs from that of the IWG. They claim that “the NIA-AA research framework uses ‘at risk’ in a much different connotation referring to asymptomatic individuals with biomarker evidence of AD as having AD but being ‘at risk’ of subsequent cognitive decline (as opposed to ‘at risk’ for AD)” (Jack *et al.*, 2018, 551). This statement ignores the fact that the IWG clearly means “at risk for *clinical* AD.”

7 Although I believe this is questionable, given Boorse’s view on dysfunction as a hierarchical goal directed concept, this is not the place to explore this point in-depth. On the other hand, the fact that they are not statistically abnormal does also count against them being dysfunctions.

8 Up till now, reduction in beta-amyloid plaques has not been shown to ameliorate the course of the clinical disease; for a recent study to this effect see Nicoll *et al.* (2019).

9 A multicausal complex dynamic model of disease does not fit very well with Boorse’s account, which focusses on disease as static pathological conditions. Given the fact that Nordenfelt’s account is much more vague about the exact condition leading to illness, it may find it easier to accommodate multi-causal, complex disease processes. Nordenfelt’s hallmark question “does this condition interfere with the person’s ability to attain vital goals” does not presume a specific type of pathophysiology, or a single causal pathway. However, I would argue that this question should be supplemented with the question: “what is the probability that this condition will lead to impairment of the ability to attain vital goals in the (near) future,” since this is most relevant from the individual’s point of view.

## References

[CIT0001] Arias, J. J., J. C. Sarrett, R. Gonzalez, and E. F. Walker. 2017. The ethics of prodromal and preclinical disease states. In The Routledge Handbook of Neuroethics, eds. L. S. M. Johnson, and K. S. Rommelfanger, 66–84. New York: Routledge Publishers.

[CIT0002] Boorse, C. 1975. On the distinction between disease and illness. Philosophy & Public Affairs 5(1):49–68.

[CIT0003] ———. 1977. Health as a theoretical concept. Philosophy of Science 44(4):542–73.

[CIT0004] ———. 1997. A rebuttal on health. In What is disease?, eds. J. M. Humber and R. F. Almeder, 3–133. Totowa, NJ: Humana Press.

[CIT0005] ———. 2014. A second rebuttal on health. Journal of Medicine and Philosophy 39(6):683–724. 25398760 10.1093/jmp/jhu035

[CIT0006] Braak, H. and E. Braak. 1991. Neuropathological stageing of Alzheimer-related changes. Acta Neuropathologica 82(4):239–59. 1759558 10.1007/BF00308809

[CIT0007] De Vreese, L. 2017. How to proceed in the disease concept debate? A pragmatic approach. Journal of Medicine and Philosophy 42(4):424–46. 28859464 10.1093/jmp/jhx011

[CIT0008] Doust, J. A., K. J. A. Bell, and P. P. Glasziou. 2020. Potential consequences of changing disease classifications. JAMA 323(10):921–2. 32031569 10.1001/jama.2019.22373

[CIT0009] Dubois, B., H. H. Feldman, C. Jacova, J. L. Cummings, S. T. Dekosky, P. Barberger-Gateau, A. Delacourte, et al. 2010. Revising the definition of Alzheimer´s disease: A new lexicon. Lancet 9(11):1118–27. 10.1016/S1474-4422(10)70223-4 20934914

[CIT0010] Dubois, B., H. H. Feldman, C. Jacova, H. Hampel, J. L. Molinuevo, K. Blennow, S. T. DeKosky, et al. 2014. Advancing research diagnostic criteria for Alzheimer’s disease: The IWG-2 criteria. Lancet Neurology 13(6):614–29. 24849862 10.1016/S1474-4422(14)70090-0

[CIT0011] Dubois, B., H. Hampel, H. H. Feldman, P. Scheltens, P. Aisen, S. Andrieu, H. Bakardjian, et al. 2016. Preclinical Alzheimer’s disease: Definition, natural history, and diagnostic criteria. Alzheimer’s & Dementia 12(3):292–323. 10.1016/j.jalz.2016.02.002 PMC6417794 27012484

[CIT0012] Giaccone, G., T. Arzberger, I. Alafuzoff, S. Al-Sarraj, H. Budka, C. Duyckaerts, P. Falkai, et al; BrainNet Europe consortium. 2011. New lexicon and criteria for the diagnosis of Alzheimer’s disease. The Lancet Neurology 10(4):298–9; author reply 300. 21435593 10.1016/S1474-4422(11)70055-2

[CIT0013] Giroux, E. 2015. Epidemiology and the bio-statistical theory of disease: A challenging perspective. Theoretical Medicine and Bioethics 36(3):175–95. 25951975 10.1007/s11017-015-9327-7

[CIT0014] Hampel, H., S. Lista, S. J. Teipel, F. Garaci, R. Nisticò, K. Blennow, H. Zetterberg, et al. 2014. Perspective on future role of biological markers in clinical therapy trials of Alzheimer’s disease: A long-range point of view beyond 2020. Biochemical Pharmacology 88(4):426–49. 24275164 10.1016/j.bcp.2013.11.009

[CIT0015] Heister, D., J. B. Brewer, S. Magda, K. Blennow, L. K. McEvoy; Alzheimer’s Disease Neuroimaging Initiative. 2011. Predicting MCI outcome with clinically available MRI and CSF biomarkers. Neurology 77(17):1619–28. 21998317 10.1212/WNL.0b013e3182343314 PMC3198979

[CIT0016] Jack, C. R. Jr, M. S. Albert, D. S. Knopman, G. M. McKhann, R. A. Sperling, M. C. Carrillo, B. Thies, and C. H. Phelps. 2011. Introduction to the recommendations from the National Institute on Aging-Alzheimer’s Association workgroups on diagnostic guidelines for Alzheimer’s disease. Alzheimer’s & Dementia 7(4S):257–62. 10.1016/j.jalz.2011.03.004 PMC3096735 21514247

[CIT0017] Jack, C. R. Jr, D. S. Knopman, W. J. Jagust, R. C. Petersen, M. W. Weiner, P. S. Aisen, L. M. Shaw, et al. 2013. Tracking pathophysiological processes in Alzheimer’s disease: An updated hypothetical model of dynamic biomarkers. Lancet Neurology 12(2):207–16. 23332364 10.1016/S1474-4422(12)70291-0 PMC3622225

[CIT0018] Jack, C. R. Jr, D. S. Knopman, W. J. Jagust, L. M. Shaw P. S. Aisen, M. W. Weiner, R. C. Petersen, and J. Q. Trojanowski. 2010. Hypothetical model of dynamic biomarkers of the Alzheimer’s pathological cascade. Lancet Neurology 9(1):119-28. 20083042 10.1016/S1474-4422(09)70299-6 PMC2819840

[CIT0019] Jack, C. R. Jr, D. S. Knopman, S. D. Weigand, H. J. Wiste, P. Vemuri, V. Lowe, K. Kantarci, et al. 2012. An operational approach to NIA-AA criteria for preclinical Alzheimer’s disease. Annals of Neurology 71(6):765–75. 22488240 10.1002/ana.22628 PMC3586223

[CIT0020] Jack, C. R., B. Bennet, and K. Blennow. 2018. The National Institute on Aging and Alzheimer’s Association Research Framework: Toward a biological definition of Alzheimer’s disease. Alzheimer’s & Dementia 14(4):535–62. 10.1016/j.jalz.2018.02.018 PMC5958625 29653606

[CIT0021] Karlawish, J. 2010. Desktop medicine. JAMA 304(18):2061–2. 21063017 10.1001/jama.2010.1624

[CIT0022] McKhann, G., D. Drachman, M. Folstein, R. Katzman, D. Price, and E. M. Stadlan. 1984. Clinical diagnosis of Alzheimer’s disease: Report of the NINCDS-ADRDA Work Group under the auspices of Department of Health and Human Services Task Force on Alzheimer’s Disease. Neurology 34(7):939–44. 6610841 10.1212/wnl.34.7.939

[CIT0023] Mirra, S. S., A. Heyman, D. McKeel, S. M. Sumi, B. J. Crain, L. M. Brownlee, F. S. Vogel, J. P. Hughes, G. van Belle, and L. Berg. 1991. The Consortium to Establish a Registry for Alzheimer’s Disease (CERAD). Part II. Standardization of the neuropathologic assessment of Alzheimer’s disease. Neurology 41(4):479–86. 2011243 10.1212/wnl.41.4.479

[CIT0024] Nicoll, J. A. R., G. R. Buckland, C. H. Harrison, A. Page, S. Harris, S. Love, J. W. Neal, C. Holmes, and D. Boche. 2019. Persistent neuropathological effects 14 years following amyloid-β immunization in Alzheimer’s disease. Brain 142(7):2113–26. 31157360 10.1093/brain/awz142 PMC6598630

[CIT0025] Nordby, H. 2006. The analytic-synthetic distinction and conceptual analyses of basic health concepts. Medicine, Health Care, and Philosophy 9(2):169–80. 16850197 10.1007/s11019-006-0002-7

[CIT0026] Nordenfelt, L. 1987. On the Nature of Health. Dordrecht, The Netherlands: D. Reidel Publishing Company.

[CIT0027] ———. 2007. The concepts of health and illness revisited. Medicine, Health Care, and Philosophy 10(1):5–10. 16955344 10.1007/s11019-006-9017-3

[CIT0028] ———. 2013. The opposition between naturalistic and holistic theories of health and disease. In Health, Illness and Disease. Philosophical Essays, eds. H. Carel and R. Cooper, 23–36. Durham, United Kingdom: Acumen.

[CIT0029] Reid, L. 2017. Truth or spin? Disease definition in cancer screening. Journal of Medicine and Philosophy 42(4):385–404. 28419307 10.1093/jmp/jhx006

[CIT0030] Richard, M. and C. Brayne. 2010. What do we mean by Alzheimer’s disease? BMJ 341(7778):865–7. 10.1136/bmj.c4670 20940218

[CIT0031] Rogers, W. A. and M. A. Walker. 2017. The line-drawing problem in disease definition. Journal of Medicine and Philosophy 42(4):405–23. 28444298 10.1093/jmp/jhx010

[CIT0032] Savva, G. M., S. B. Wharton, P. G. Ince, G. Forster, F. E. Matthews, and C. Brayne. 2009. Age, neuropathology, and dementia. New England Journal of Medicine 360(22):2302–9. 19474427 10.1056/NEJMoa0806142

[CIT0033] Scheltens, P., K. Blennow, M. M. B. Bretelaar, B. de Strooper, G. B. Frisoni, S. Salloway, and W. M. Van der Flier. 2016. Alzheimer’s disease. Lancet 388(10043):505–17. 26921134 10.1016/S0140-6736(15)01124-1

[CIT0034] Schermer, M. H. N. and E. Richard. 2019. On the reconceptualization of Alzheimer’s disease. Bioethics 33(1):138–45. 30303259 10.1111/bioe.12516 PMC6585806

[CIT0035] Schwartz, P. H. 2007. Defining dysfunction: Natural selection, design, and drawing a line. Philosophy of Science 74(3):364–85.

[CIT0036] ———. 2008. Risk and disease. Perspectives in Biology and Medicine 51(3):320–34. 18723938 10.1353/pbm.0.0027

[CIT0037] ———. 2014. Small tumors as risk factors not disease. Philosophy of Science 81(5):986–98.

[CIT0038] ———. 2017. Progress in defining disease: Improved approaches and increased impact. Journal of Medicine and Philosophy 42(4):485–502. 28859465 10.1093/jmp/jhx012

[CIT0039] Sperling, R., E. Mormino, and K. Johnson. 2014. The evolution of preclinical Alzheimer’s disease: Implications for prevention trials. Neuron 84(3):608–22. 25442939 10.1016/j.neuron.2014.10.038 PMC4285623

[CIT0040] Sperling, R. A., P. S. Aisen, L. A. Beckett, D. A. Bennett, S. Craft, A. M. Fagan, T. Iwatsubo, et al. 2011. Toward defining the preclinical stages of Alzheimer’s disease: Recommendations from the National Institute on Aging-Alzheimer’s Association workgroups on diagnostic guidelines for Alzheimer’s disease. Alzheimer’s & Dementia 7(3):280–92. 10.1016/j.jalz.2011.03.003 PMC3220946 21514248

[CIT0041] Sperling, R. A., J. Karlawish, and K. A. Johnson. 2013. Preclinical Alzheimer disease - the challenges ahead. Nature Reviews Neurology 9(1):54–8. 10.1038/nrneurol.2012.241 PMC3643203 23183885

[CIT0042] Sweeney, M. D., A. Montagne, A. P. Sagare, D. A. Nation, L. S. Schneider, H. C. Chui, M. G. Harrington, et al. 2019. Vascular dysfunction – the disregarded partner of Alzheimer’s disease. Alzheimer’s & Dementia 15(1):158–67. 10.1016/j.jalz.2018.07.222 PMC6338083 30642436

[CIT0043] Walker, M. J. and W. A. Rogers. 2018. A new approach to defining disease. Journal of Medicine and Philosophy 43(4):402–20. 29986065 10.1093/jmp/jhy014

[CIT0044] Wolfsgruber, S., A. Polcher, A. Koppara, L. Kleineidam, L. Frölich, O. Peters, M. Hüll, et al. 2017. Cerebrospinal fluid biomarkers and clinical progression in patients with subjective cognitive decline and mild cognitive impairment. Journal of Alzheimer’s Disease 58(3):939–50. 10.3233/JAD-161252 28527210

